# Autoimmunity and microbiota

**DOI:** 10.1186/1479-5876-9-S2-I13

**Published:** 2011-11-23

**Authors:** Alexander Chervonsky

**Affiliations:** 1 The University of Chicago, Chicago, USA

## 

Environment (physical insults, toxins, nutrition and microorganisms) influences development of autoimmune diseases. Pathogenic, pathobiotic and commensal microorganisms contribute to disease progression in genetically susceptible hosts. Germ-free animals or defined-microbiota colonized animals are valuable tools for understanding microbial influences on autoimmunity, especially if they could be genetically modified.

Type 1 diabetes (T1D) susceptible NOD mice were used as an autoimmunity model to test the role of microbiota in disease development and we have determined that microbiota manipulates innate immune system affecting T1D progression.In addition, we found that metabolic changes affecting T1D pathogenesis work indirectly through microbiota. Thus, genetics, microbiota, metabolism and likely dietary challenges, can all affect development of autoimmune diseases.

